# Characterization of a New Powdery Mildew Resistance Gene on Chromosome 1R from Hexaploid Triticale Transferred to Wheat

**DOI:** 10.3390/plants15030410

**Published:** 2026-01-29

**Authors:** Yujie Luo, Chengzhi Jiang, Li Li, Tingting Jiang, Jessy Yee Ting Tan, Aly Boro, Ennian Yang, Guangrong Li, Zujun Yang

**Affiliations:** 1 School of Life Science and Technology, University of Electronic Science and Technology of China, Chengdu 610054, China; 202321140401@std.uestc.edu.cn (Y.L.); 202311140626@std.uestc.edu.cn (C.J.); 202421140402@std.uestc.edu.cn (L.L.); 202421140401@std.uestc.edu.cn (T.J.); 202424140101@std.uestc.edu.cn (J.Y.T.T.); 202514140102@std.uestc.edu.cn (A.B.); 2 Crop Research Institute, Sichuan Academy of Agricultural Sciences, Chengdu 610066, China; yangennian@126.com

**Keywords:** rye, wheat, ND-FISH, powdery mildew resistance

## Abstract

Powdery mildew, caused by *Blumeria graminis* f. sp. *tritici*, is a highly destructive disease affecting wheat in most growing regions worldwide. The most effective strategy for combating this disease is through the exploitation of novel and durable resistance genes derived from the relatives of wheat. Rye (*Secale cereale* L.) has been extensively hybridized with both tetraploid and hexaploid wheats and represents a valuable genetic resource for enhancing resistance and tolerance to both biotic and abiotic stresses. In this study, two novel 1R (1D) substitution lines, R156 and R189, derived from hexaploid triticale lines Yukuri and T4915, respectively, were comprehensively characterized using non-denaturing fluorescence in situ hybridization (ND-FISH) and immunofluorescence. To physically map the 1R-derived powdery mildew resistance gene from Yukuri, 3485 M_1_-M_3_ plants from the cross between R156 and susceptible wheat cultivar MY11 were studied by ND-FISH using multiple probes. A cytological bin map for Yukuri chromosome 1R was constructed using 105 molecular markers. Resistance evaluation combined with molecular mapping revealed that the novel resistance locus resides in bin 1RS-4, corresponding to the 58.60–109.28 Mb genome region of Lo7 rye chromosome 1R. Thus, these newly developed wheat–rye 1R translocation and deletion lines are expected to serve as valuable genetic resources for breeding powdery mildew resistant wheat cultivars.

## 1. Introduction

Common wheat (*Triticum aestivum* L.) is a staple cereal cultivated worldwide, constituting a primary nutritional source for over one-third of the global population [1,2]. Powdery mildew, caused by the biotrophic fungal pathogen *Blumeria graminis* f. sp. *tritici* (*Bgt*), is a major wheat disease prevalent across most production regions [3,4]. Breeding resistant cultivars is widely considered the most cost-effective and environmentally sustainable strategy for disease control. To date, 71 powdery mildew resistance genes (*Pm*) have been formally cataloged, several of which have been deployed in wheat breeding programs [5]. Substantial research has underscored the value of wheat relatives as reservoirs of *Bgt* resistance, exemplified by genes such as *Pm21* and *Pm67* from *Dasypyrum villosum* [6,7] and *Pm13* and *Pm66* from *Ae. longissima* [8,9]. These findings highlight the significant contribution of wheat relatives as valuable sources of *Pm* resistance genes for wheat breeding programs aimed at producing commercially successful cultivars.

Rye (*Secale cereale* L., 2n = 2x = 14, RR) has been widely utilized as a significant germplasm resource for improving disease resistance, environmental adaptability, and yield in wheat improvement programs [10,11]. The introduction of diversified rye genes into wheat can increase genetic variation and result in novel germplasm with significant breeding potential. Hexaploid triticale (*×Triticosecale* Wittmack, 2n = 6x = 42, AABBRR), synthesized artificially by hybridizing tetraploid wheat with rye, serves as a bridge species that enables, by subsequent hybridizations with wheat, the efficient development of wheat–rye substitution or addition lines. For instance, Yan et al. [12] developed a novel wheat–rye 2R (2D) substitution line, YT9, by crossing the hexaploid triticale line 09R1-16 with the wheat breeding line PB9, which conferred a high level of resistance to powdery mildew. Moreover, the 6RL chromosome arm from triticale line 09R2-121 was shown to confer powdery mildew resistance [13], and the 4RL chromosome arm derived from triticale cultivar Soreneo also contributes resistance to this disease [14]. However, some rye-derived resistance genes have been overcome by the emergence of virulent mutant pathogens, leading to a severe reduction in wheat production in certain regions [15].

Rye chromosome 1R has been extensively and successfully utilized in wheat resistance breeding. The 1RS segment derived from Petkus rye confers resistance to several major wheat diseases, including leaf rust (*Lr26*), stripe rust (*Yr9*), stem rust (*Sr31*), and powdery mildew (*Pm8*), and has been widely used around the world in commercial agriculture [16]. Subsequently, a novel translocation, 1RS.1AL, identified in Amigo wheat, was found to carry the stem rust resistance gene, *Sr1RS^Amigo^*, and the powdery mildew resistance gene, *Pm17*, which is allelic to *Pm8* [17]. In addition, recent efforts have focused on transferring new resistance factors from diverse 1R sources. For example, Li et al. [18] developed three 1RS.1BL translocation lines through crosses between wheat cultivar Mianyang11 and Aigan rye, all of which exhibited high resistance to stripe rust. Similarly, nine novel T1RS.1AL translocation lines derived from wheat Chuannong25 (CN25) and Chinese native rye cv. Qinling showed strong resistance to both stripe rust and powdery mildew [19]. However, the evolving virulence of wheat pathogens has led to the loss of effectiveness of some of these resistance genes, presenting ongoing challenges to wheat breeding. To address the crisis of rapidly diminishing stocks of effective disease resistance, it is essential to continue to identify and introduce new resistance sources from diverse rye genotypes into elite wheat germplasm and transfer that resistance to wheat chromosomes.

Fluorescence in situ hybridization (FISH) with synthetic oligonucleotide probes offers advantages over traditional FISH, including higher throughput and greater probe stability. In the present study, we employed ND-FISH to characterize two wheat–rye derivative lines, A156 and A189, which derived from the hexaploid triticale lines Yukuri and T4915, respectively. Both lines demonstrated high resistance to powdery mildew under natural field conditions. The objectives of this study were to (1) characterize the chromosome compositions of A156 and A189 using ND-FISH and Immuno-FISH, (2) investigate comparative sequence variations of the resistance loci among different rye 1R chromosomes, and (3) evaluate the resistance to powdery mildew and physically map the resistance gene(s) in lines derived from A156. This work aims to provide novel and potentially valuable germplasm resources for breeding disease-resistant wheat varieties and insights into the location and structure of the *Pm* resistance genes therein.

## 2. Results

### 2.1. Chromosomal Composition of Lines A156 and A189

**Figure 1:**
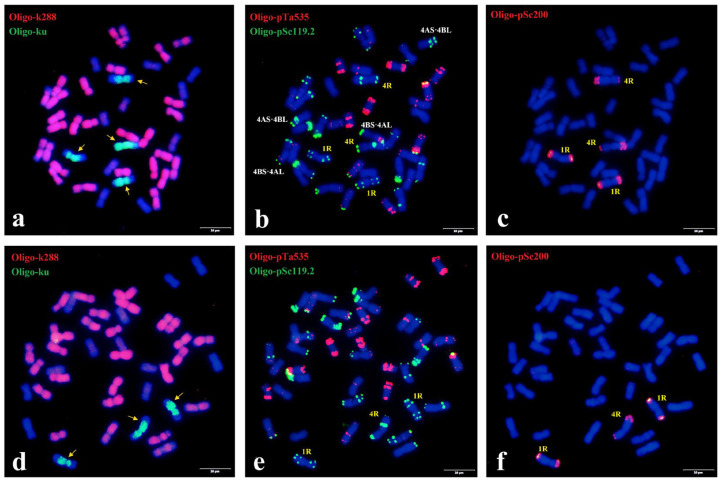
Karyotyping of mitotic metaphases of A156 (**a**–**c**) and A189 (**d**–**f**) by sequential ND-FISH. Sequential ND-FISH by probes (**a**,**d**) Oligo-k288 (red) + Oligo-ku (green), (**b**,**e**) Oligo-pTa535 (red) + Oligo-pSc119.2 (green), and (**c**,**f**) Oligo-pSc200 (red), respectively. Rye chromosomes are indicated by yellow arrows. Scale bars, 10 μm.

Sequential ND-FISH with probes Oligo-k288, Oligo-ku, Oligo-pSc119.2, Oligo-pTa535 and Oligo-pSc200 was utilized to characterize the chromosome constitutions of A156 and A189 (Figure 1a–f). Rye genome-specific probe Oligo-ku showed that A156 carried 4 rye chromosomes, wheat AABB genome-specific probe Oligo-k288 hybridized to 28 chromosomes, while the remaining 10 chromosomes were of the D-genome. Sequential ND-FISH with probes Oligo-pSc119.2, Oligo-pTa535 and Oligo-pSc200 confirmed that A156 had 38 wheat chromosomes and chromosomes 1D and 4D were substituted by the rye chromosomes 1R and 4R. Moreover, two pairs of reciprocal Robertsonian translocation chromosomes 4AS·4BL and 4BS·4AL were identified in A156 (Figure 1a–c). In addition, A189 carried 43 chromosomes, of which 3 hybridized with probes Oligo-ku and Oligo-pSc200. ND-FISH using probes Oligo-pTa535 and Oligo-pSc119.2 showed that A189 had two 1R chromosomes and one 4R chromosome (Figure 1d–f). Based on the comprehensive cytogenetic analyses, A156 was characterized as a wheat–rye double disomic substitution 1R (1D) + 4R (4D) line, and A189 was characterized as a 1R (1D) substitution and 4R monosomic addition line.

### 2.2. Immuno-FISH Analysis of Two 1R (1D) Substitution Lines

To separate and transfer the different 1R chromosomes from A156 and A189, we crossed both lines A156 and A189 with wheat cultivar MY11. A total of 876 F2 plants were screened by ND-FISH using probes Oligo-pSc119.2, Oligo-pTa535 and Oligo-pSc200. Twenty-one and twenty-six 1R (1D) substitution lines were developed from A156 and A189, respectively (Figure S1). Subsequently, we selected two stable 1R substitution lines R156 (from A156) and R189 (from A189) for immunofluorescence.

**Figure 2:**
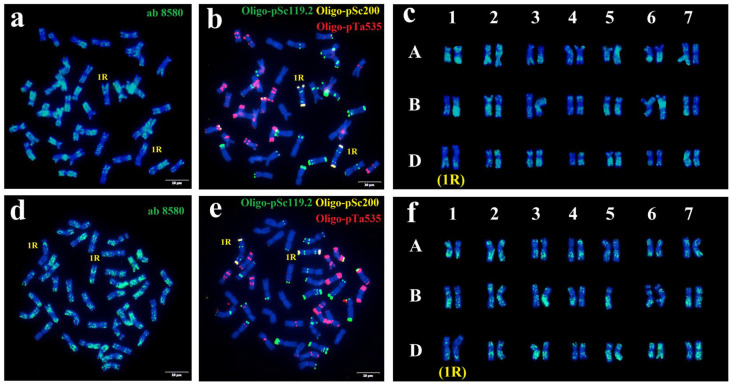
The anti-methylation location and ND-FISH of mitotic metaphase of 1R (1D) substitution lines R156 (**a**–**c**) and R189 (**d**–**f**). Immunostaining with anti-methylation ab 8580 (green) (**a**,**d**) and sequential ND-FISH by Oligo-pSc119.2 (green) + Oligo-pTa535 (red) + Oligo-pSc200 (red) (**b**,**e**). (**c**,**f**) The cut and pasted chromosomes of anti-methylation signals. Scale bars, 10 μm.

Immuno-FISH analysis of anti-CENH3 showed that all wheat chromosomes and rye 1R chromosomes had the CENH3 signals in the centromeric regions, indicating that the 1R chromosomes may have accumulated enough content of the CENH3 protein in the centromeric regions during mitosis for reliable chromosome transmission (Figure S2). Anti-phosphorylation AG3875 and AG3665 signals were also deposited in the centromeric positions of all wheat and rye 1R chromosomes at the metaphase of mitosis but were absent at the mitotic interphase (Figure S3). Moreover, immuno-FISH analysis of anti-methylation ab8580 and ND-FISH revealed that the methylation sites were unevenly distributed across all chromosomes in two 1R (1D) substitution lines. The methylation levels across the three subgenomes of wheat were similar. Meanwhile, the introduction of different 1R chromosomes had a relatively minor effect on wheat methylation accumulation. Notably, two 1R chromosomes in one metaphase exhibited different methylation patterns; for instance, the short arm of one 1R displayed a distinctly strong methylation signal, suggesting potential differences in the expression levels between the two 1R chromosomes (Figure 2a–f). This differential methylation was also observed in the 1RS.1BL translocation line (Figure S4).

### 2.3. Alignment of Yr9/Pm8 Gene-Specific Primer (GSP) Sequences

**Figure 3:**
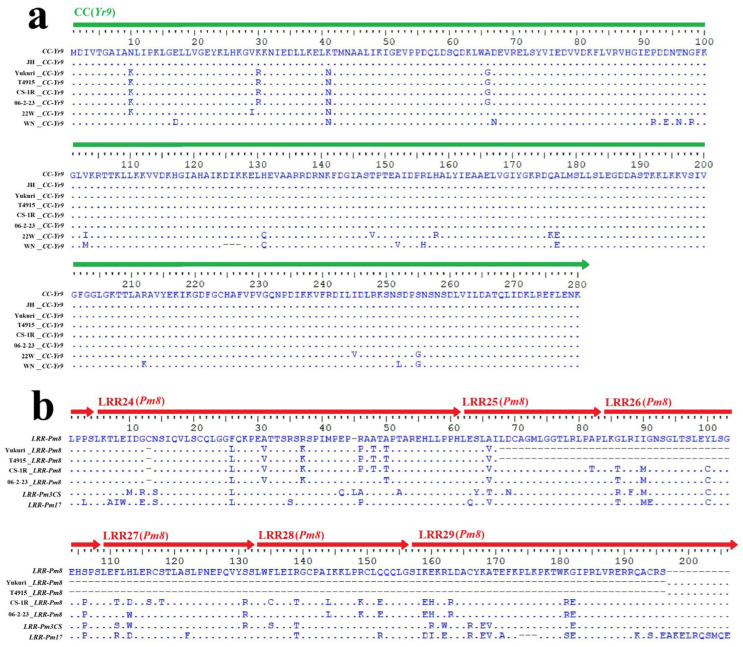
Alignment of GSP sequences of *Yr9* and *Pm8* in the representing 1R chromosomes. (**a**) The sequences from top to bottom are CC-*Yr9*, JH_CC-*Yr9*, Yukuri_CC-*Yr9*, T4915_CC-*Yr9*, CS-1R_CC-*Yr9*, 06-2-23_CC-*Yr9*, 22W_CC-*Yr9* and WN_CC-*Yr9*, which are originated from 1R of Lo7, JH rye, Yukuri, T4915, Imperial rye, 06-2-23, Currency and Weining rye, respectively. (**b**) The sequences from top to bottom are LRR-*Pm8*, Yukuri_LRR-*Pm8*, T4915_LRR-*Pm8*, CS-1R_LRR-*Pm8* and 06-2-23_LRR-*Pm8*, which are originated from 1R of Lo7, Yukuri, T4915, Imperial rye and 06-2-23, respectively. The sequences LRR_*Pm3CS* from CS and LRR_*Pm17* from Insave rye were used as controls. The CC and LRR domains are indicated by green and red bars above, respectively.

The cloned *Pm8* and *Yr9* genes, located on chromosome 1RS, corresponded to the SECCE1Rv1G0001880.1 and SECCE1Rv1G0003760.1 sequences of the Lo7 genome, respectively. The amplification products of *Yr9* gene-specific primers (GSPs) showed that 1R from Yukuri, T4915 and other five rye accessions may contain *Yr9* or its allele (Figure S5). Then we cloned the GSP-derived PCR products of the seven rye lines which contained a large part of CC domain sequences of the *Yr9* gene. Six sequences were 842 bp in length, while WN_CC-*Yr9* was only 833 bp (Figure 3a). Protein primary sequence alignment showed that only 23 (8.2%) amino acid sites had changes compared with CC-*Yr9*, of which 15 sites showed 87.5% homology. Four sequences (Yukuri_CC-*Yr9*, T4915_CC-*Yr9*, CS-1R_CC-*Yr9* and 06-2-23_CC-*Yr9*) contained the same primary protein sequence; however, all sequences contained 1-3 SNPs at different sites. Subsequently, we cloned the PCR-derived products using the *Pm8* GSP sequences for four 1R chromosomes, all of which contained the small part of LRR domain sequences of the *Pm8* gene. The four sequences were all 585 bp in length and exhibited a 3 bp deletion compared with the GSP sequence of LRR-*Pm8* (Figure 3b). Protein primary sequence alignment showed that 28 (13.6%) amino acid sites had changes. Notably, Yukuri_LRR-*Pm8* and T4915_LRR-*Pm8* were pseudogenes due to the in-frame stop codon that occurred at the LRR25 domains. This finding indicates that the functional *Pm8* gene is absent or non-functional in these lines, suggesting that the observed powdery mildew resistance is conferred by a different, novel locus. The results indicate that the two 1R chromosomes in Yukuri and T4915 which contained a novel stripe rust and powdery mildew resistance locus were different from other 1R chromosomes based on their nucleotide sequences.

### 2.4. Chromosome Variations in M_1_ Population of a 1R (1D) Substitution Line from Yukuri

To generate structural variants of the 1R^Yukuri^ chromosome, line R156 was crossed with the common wheat cultivar MY11. The resulting F1 seeds were subjected to γ-irradiation at a dose of 200 Gy (1.00 Gy/min). A total of 173 M_0_ grains were germinated and produced selfed progenies. Karyotype analysis was then performed on 1823 M_1_ plants using oligonucleotide probes Oligo-ku, Oligo-k288, Oligo-pSc119.2, Oligo-pTa535 and Oligo-pSc200. Chromosomal variations were detected in 303 plants (18.6%), among which 51 distinct types of aberrations involving the 1R chromosome were identified (Figure S6). The 51 aberrations were categorized into whole-arm translocation lines (five plants), small-segment translocation lines (21 plants), large-segment translocation lines (18 plants) and deletion lines (seven plants). The identified 1R deletion and translocation lines were subsequently backcrossed to MY11 to facilitate the selection of homozygous progenies for physical mapping.

### 2.5. Identification of Wheat–Rye 1R Deletion and Translocation Lines

**Figure 4:**
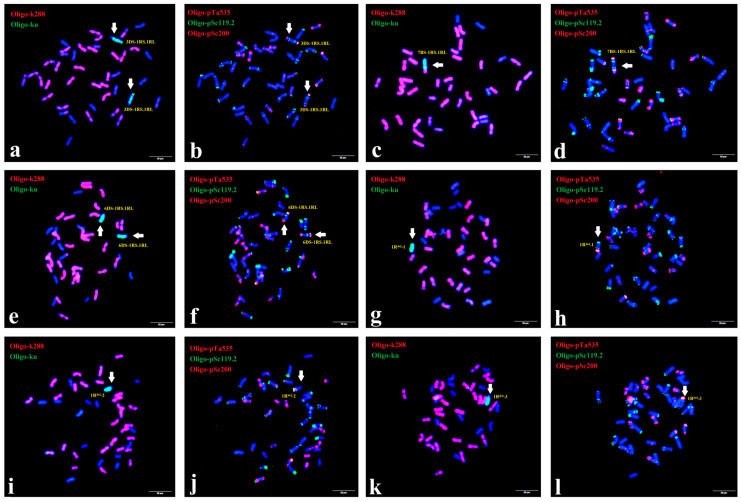
FISH of wheat–rye 1R aberration lines X814 (3DS-1RS.1RL) (**a**,**b**), X330 (7BS-1RS.1RL) (**c**,**d**), X617 (6DS-1RS.1RL) (**e**,**f**), X383 (1R^del^-1) (**g**,**h**), X503 (1R^del^-2) (**i**,**j**) and X342 (1R^del^-3) (**k**,**l**) from F_2_ progenies. The probes were Oligo-k288 (red)+ Oligo-ku (green) (**a**,**c**,**e**,**g**,**i**,**k**) and Oligo-pTa535 (red) + Oligo-pSc119.2 (green) +Oligo-pSc200 (red) (**b**,**d**,**f**,**h**,**j**,**l**). The arrows indicate the translocation or deletion chromosomes. Bars, 10 μm.

A total of 786 F_2_ progenies derived from those 1R aberrations were characterized by ND-FISH with multiple probes. Six plants were characterized to have a single aberration type involving the 1R chromosome. Among them, three were translocations, and three were deletions. The three translocations all involved large fragments of the rye chromosome (3DS-1RS.1RL, 7BS-1RS.1RL and 6DS-1RS.1RL). As shown in Figure 4a,b, line X814 had 42 chromosomes, including 40 wheat chromosomes and 2 1R translocations. Sequential ND-FISH with probes Oligo-ku + Oligo-k288 and Oligo-pSc119.2 + Oligo-pTa535 indicated that the wheat segment of this new translocation originated from chromosome 3D. This translocation chromosome in X814 is thus named 3DS-1RS.1RL. Similarly, line X330 contained a single 1R translocation, named 7BS-1RS.1RL (Figure 4c,d). In addition, a smaller-segment translocation between the D-genome and chromosome 1R was detected in line X617 using probes Oligo-ku + Oligo-k288 and Oligo-pSc119.2 + Oligo-pTa535. These probes displayed strong green signals at the terminal regions, indicating that a segment of 6DS had fused with the terminus of chromosome 1R, resulting in the translocated chromosome designated as 6DS-1RS.1RL (Figure 4e,f). Regarding deletions, line X383 carried a single 1R^del−1^ chromosome. Sequential ND-FISH with probes Oligo-pSc119.2 + Oligo-pTa535 + Oligo-pSc200 on the same metaphase cell revealed that the breakpoint lay between the strong signals of Oligo-pSc119.2 and Oligo-pSc200 (Figure 4g,h). Similarly, two other distinct 1R deletions (1R^del−2^ and 1R^del−3^) are presented in Figure 4i–l. Following self-pollination, these 1R structural variants were stably transmitted to subsequent generations, enabling their use for the precise mapping of agronomically important genes.

### 2.6. Construction of a Physical Map of 1R Chromosomes

A total of 71 IT (intron-targeting) primers and 92 SSR primers were used to screen 1R-specific PCR markers, and 105 (42 IT primers and 63 SSR primers) of them amplified bands from Yukuri, compared with wheat cultivar MY11. Since the background wheat cultivar used to develop Yukuri was unavailable, we deduced that these non-MY11 bands were specific to the 1R chromosome from Yukuri (a) because all primer sequences corresponded to the 1R chromosome of the *Secale*_Lo7 reference genome sequence and (b) due to the linkage of the presence or absence of a PCR-derived amplification with the cytological presence or absence of a 1R chromosome, respectively, in the individual seedlings under study. A total of 45 and 60 markers were distributed on chromosome arms 1RS and 1RL, respectively. All the primer sequences were used for the BLAST searching of the *Secale*_Lo7 reference genome sequences by B2DSC web sites to construct a physical map of the 1R chromosome. Meanwhile, the 1R chromosome from Yukuri exhibited highly polymorphic amplifications compared with other rye accessions (Figure S7), indicating that substantial sequence divergences have accumulated among the rye 1R chromosomes. Notably, three 1R-derived lines from F_2_ progenies had lost the signals for probe Oligo-pSc200; however, 1R-specific markers failed to detect the breakpoints in these lines. We then developed five 1R-specific primers by screening single-copy sequences from 1R, which revealed extensive polymorphisms in the terminal regions of 1RS (Figure S8).

**Figure 5:**
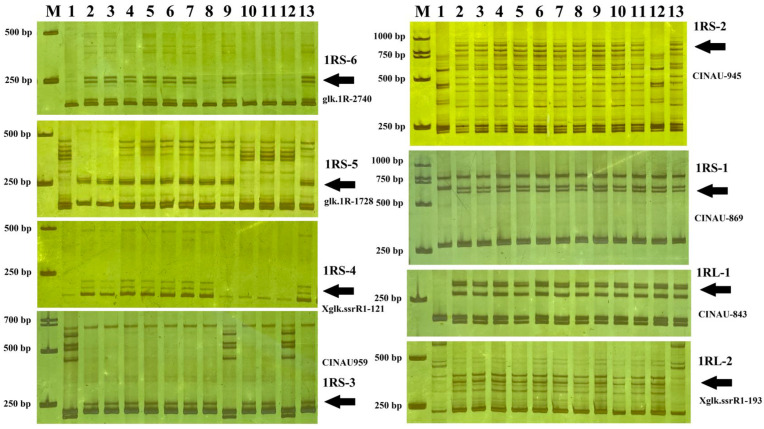
PCR amplification results of representative 1R-specific markers in the six translocation and deletion lines. 1, MY11; 2, Yukuri; 3, T4915; 4, A156; 5, A189; 6, R156; 7, R189 8, X814; 9, X330; 10, X617; 11, X383; 12, X503; 13, X342. Arrows point to chromosome 1R-specific bands.

**Figure 6:**
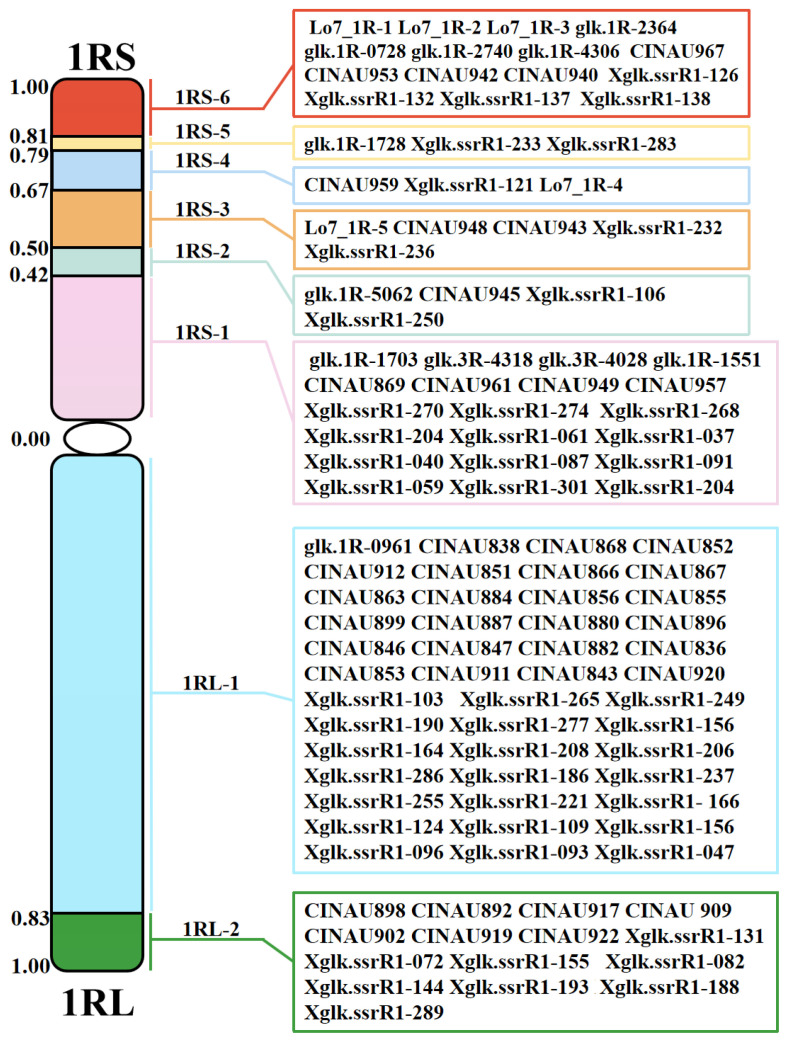
A cytological bin map of 1R chromosome showing 8 bins by the genotyping of 6 rearrangement lines using 108 1R diagnostic markers. The 6 bins of the 1R short arm were designated as 1RS-1 to 1RS-6, and the 2 bins of the 1R long arm were designated as 1RL-1 to 1RL-2. IT and SSR markers were assigned to different chromosomal bins.

Subsequently, all the 1R-specific markers were used to determine the breakpoints of the twelve homozygous 1R translocation and deletion lines. Among them, five aberration lines (X814, X330, X617, X383 and X503) included six different 1RS chromosomal bins. The remaining one deletion line X342 dissected 1RL into two bins. The representative markers for different breakpoint types are shown in Figure 5. Thus, we constructed the physical map of the 1R chromosome from Yukuri (Figure 6), and the 108 markers could be anchored into eight physical regions. We assigned 15, 3, 3, 5, 4, 19, 44, and 15 markers to the chromosomal bins of 1RS-6, 1RS-5, 1RS-4, 1RS-3, 1RS-2, 1RS-1, 1RL-1, and 1RL-2, respectively. The constructed 1R physical map was further used to locate the powdery mildew resistance locus to a specific chromosomal region.

### 2.7. Responses of A156 and Derived Lines to Powdery Mildew

In order to evaluate the contribution of the two 1R (1D) substitution lines R156 and R189 to powdery mildew resistance, we phenotyped the lines carrying 1R chromosomes as well as their parental lines under exposure to *Blumeria graminis* f. sp. *tritici* isolates at both seedling and adult plant stages. As shown in Figure S9, A156 and A189 were highly resistant to powdery mildew (IT = 0) at both seedling and adult stages, while MY11 was highly susceptible (IT = 4). Among their progenies, plants carrying the 1R chromosomes were highly resistant to powdery mildew (IT = 0), whereas plants without the 1R chromosomes were susceptible to powdery mildew (IT = 3). The results indicate that two 1R chromosomes from A156 and A189 contribute to the enhanced all-stage resistance (ASR) to powdery mildew.

**Figure 7:**
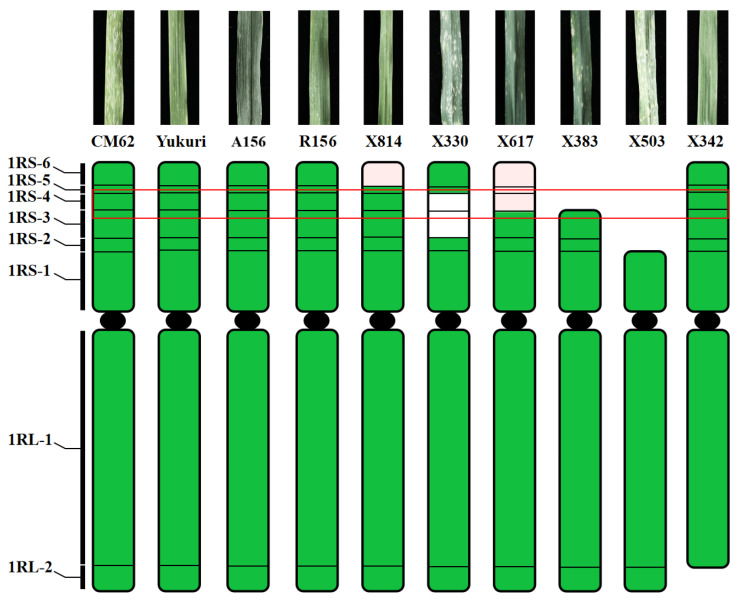
Physical mapping of the powdery mildew resistance gene using six 1R aberration lines. Green color indicates chromosome segments of 1R, and pink represents wheat chromosome fragments. The red box indicates the physical region of the *Pm* locus.

We subsequently evaluated the adult stage reactions of the six 1R aberration lines. Four lines (X330, X617, X503 and X383) were all powdery mildew susceptible, and two lines (X814 and X342) containing bin 1RS-4 were all powdery mildew resistant like the parental line (Table S1). Thus, we further allocated the powdery mildew resistant locus to bin 1RS-4, which corresponded to 58.60–109.28 Mb in the reference *Secale*_Lo7 genome sequence of 1R (Figure 7). According to the gene annotation, a total of 410 genes (SECCE1Rv1G0009900.1-SECCE1Rv1G0013990.1) have been identified in this region. Among them, five genes were annotated as LRR disease resistance protein or receptor-like kinase protein. RNA-seq data revealed that three of these genes were expressed in Yukuri following powdery mildew infection (Table S2).

## 3. Discussion

As a crucial gene pool for wheat genetic improvement, rye possesses numerous disease resistance genes that can be effectively integrated into wheat breeding programs to enhance cultivar productivity [20]. Hexaploid triticale is produced by hybridizing tetraploid wheat with diploid rye, followed by chromosome doubling in the F_1_ generation. This variety exhibits traits that are intermediate between wheat and rye and retains rye’s advantageous characteristics, such as cold tolerance, drought resistance, and pest and disease resistance [21,22]. In this study, using hexaploid triticale lines Yukuri and T4915, we developed two wheat–rye derivative lines, A156 and A189, which exhibited different powdery mildew resistance patterns. Subsequently, we selected two new wheat–rye 1R (1D) substitution lines by ND-FISH with multiple Oligo probes. This study highlights a novel powdery mildew resistance locus in Yukuri-derived 1R (1D) substitution line R156 and reports the development of small wheat-1R translocation lines with improved resistance, which represent potentially valuable resources for wheat disease resistance breeding.

Fluorescence *in situ* hybridization (FISH) and genomic *in situ* hybridization (GISH) are convenient and efficient techniques for chromosome identification in bread wheat and related species, facilitating the detection of the transfer of chromosome segments carrying elite genes from rye into wheat [23]. Using GISH with rye total genomic DNA as a probe, Li et al. [2] identified six 6R translocation and deletion lines and mapped the stripe (yellow) rust resistance gene *Yr83* to the distal bin of FL 0.73–1.00 on chromosome 6RL. Subsequently, a series of structural variants of 2R chromosomes were characterized by GISH, and a growth-stage-dependent powdery mildew resistance gene, *PmYT9*, was fine-mapped to a 14.55 Mb interval on 2RL [12]. The advent of non-denaturing FISH (ND-FISH) combined with synthetic oligonucleotide (Oligo) probes has revolutionized high-throughput detection of alien chromatin in wheat [24]. For instance, Fu et al. [25] developed probe Oligo-1162 based on SLAF-seq data of cultivated rye cv. Kustro, which produced specific signals along the entire length of rye chromosomes in octoploid triticale. In addition, a new set of Oligo probes was developed to establish a high-resolution karyotype of rye chromosomes in triticale Yukuri [26]. In the present study, we characterized two wheat–rye 1R (1D) substitution lines derived from Yukuri and T4915 using Oligo probes Oligo-ku, Oligo-pSc119.2, and Oligo-pSc200. Oligo-pSc200 produced strong hybridization signals in the terminal regions of the long and short arms, enabling rapid and precise identification of 1R segments in subsequent irradiated generations. Moreover, multiple Oligo probes successfully distinguished the 1R chromosomes from different accessions (Figure S10). Collectively, our results demonstrate that ND-FISH is highly effective for characterizing rye chromosomes based on polymorphic hybridization patterns and can be used to accurately identify introgressed rye segments in a wheat background.

Chromosome 1R has historically played an important role in wheat breeding programs. Owing to its high recombination rate, numerous valuable 1R translocation lines have been generated and deployed in resistance breeding. In the last century, the 1RS arms from Petkus rye and Insave rye were introduced into wheat via T1RS·1BL and T1RS·1AL translocations, respectively, incorporating resistance genes such as *Yr9*, *Sr31*, *Lr26*, *Pm8*, and *Pm17* into wheat, and eventually released as cultivars [16]. In recent years, several new rye 1R sources have been transferred into the wheat genome. However, polymorphisms of infection reactions to pathogens remain the primary trait used to distinguish 1R lines of different origins. Further cytological and molecular characterization of these 1R chromosomes is essential to understanding the genetic differentiation and diversity among various rye sources. Annamaria et al. [27] evaluated six SSR and STS markers across 20 rye cultivars of diverse origins and identified three polymorphic markers in Kisvárdai Alacsony, Kriszta, and Porto. Wang et al. [26] used SDS-PAGE to analyze seed storage proteins in different wheat–rye 1R materials, and the unique glutenin subunit profile of triticale cultivar Yukuri suggested a distinctly different rye origin compared with other reported triticale varieties. Moreover, it is difficult to distinguish different 1R chromosomes by traditional cytological methods because of the limitation of high-precision probes. For example, Yang et al. [28] assessed genetic diversity among 153 wheat lines from CIMMYT and Chinese core germplasm using ND-FISH with probes Oligo-pSc119.2-1, Oligo-pTa535-1, and Oligo-(GAA)_7_. Fourteen 1RS·1BL translocation lines showed identical ND-FISH patterns. In our study, we contrasted the hybridization patterns of different 1R chromosomes by using multiple Oligo probes and characterized the polymorphisms by intron-targeting (IT) markers. Meanwhile, the immunostaining for the metaphase cells of wheat–rye derivatives R156 and R189 exhibited differential methylation patterns (Figure 2), indicating the genetic distinctness of the two novel 1R (1D) substitution lines developed in our study.

Wang et al. [29] analyzed *Yr9* allelic sequences across 32 representative 1RS·1BL translocation lines and identified nine SNPs in the CC domain that resulted in four amino acid changes; however, these alterations did not compromise the disease resistance function. In our study, we cloned seven CC-*Yr9* allelic sequences from different 1R chromosomes. Four of them contained identical amino acid changes to those mentioned above (98.6% sequence identity), while the sequence from the 1Ra chromosome was identical to that of CC-*Yr9* (Figure 3). In contrast, 22W_CC-*Yr9* (from Currency triticale) and WN_CC-*Yr9* (from Weining rye) showed 11 and 15 amino acid changes compared with CC-*Yr9*, corresponding to sequence identities of 96.1% and 93.6%, respectively, indicating considerable divergence in these two 1R chromosomes. These findings suggest that multiple *Yr9* resistance alleles may exist in rye 1R chromosomes, and whether the observed amino acid variations affect gene function requires further investigation. Notably, LRR-*Pm8* allelic sequences share only 85.7% amino acid sequence identity. Moreover, Yukuri_LRR-*Pm8* and T4915_LRR-*Pm8* were identified as pseudogenes due to the presence of in-frame stop codons within the LRR25 domains. These observations imply that different resistance genes have likely undergone independent evolutionary paths under varying environmental pressures and domestication histories. Meanwhile, although *Pm8* has lost its resistance to prevalent *Bgt* pathotypes, the rapid evolution of rye 1R chromosomes continues to drive contemporary breeders to utilize rye germplasm resources in response to fast-evolving powdery mildew pathogens.

Powdery mildew is a devastating disease that severely compromises yield and quality of wheat worldwide. To date, 10 *Pm* genes have been reported on homoeologous group 1 chromosomes from diversified wheat genotypes, including *Pm3*, *Pm8*, *Pm10*, *Pm17*, *Pm24*, *Pm25*, *Pm28*, *Pm32*, *Pm39* and *Pm67* [30]. Among these, *Pm3*, *Pm10*, *Pm24*, *Pm25*, *Pm28* and *Pm39* were derived from common wheat [31,32,33,34]; *Pm67* from *D. villosum* [7]; *Pm32* from *Ae. speltoides* [35]; and *Pm8* and *Pm17*, which were proven to be homoeologous genes of *Pm3*, were derived from rye. However, *Pm8* and *Pm17* have lost their effectiveness against *Bgt* isolates currently common in many wheat production regions [36,37]. Recently, a new powdery mildew resistance gene, *PmSESY*, derived from the rare rye wild species *S. sylvestre*, was mapped to the 1.88 cM genetic interval at the end of the 1RL chromosome [38]. Ren et al. developed five stripe rust- and powdery mildew resistant 1BL.1RS translocations from Chinese rye “Baili”, which also showed significantly better yield traits in the field [39]. In our study, we also located the powdery mildew resistance gene on 1RS, while wheat with chromosome arm 1RL was susceptible to powdery mildew. Moreover, we selected six 1R deletion and translocation lines from M_1_-M_3_ generations and mapped the powdery mildew resistance gene in bin 1RS-4, covering the corresponding region of 58.60–109.28 Mb in the reference *Secale*_Lo7 genome sequence, in which only five disease resistance-related genes were predicted (Table 1). Notably, the mapped resistance locus in bin 1RS-4 is distinct from the genomic position of the canonical *Pm8* gene on 1RS. While we successfully mapped the novel powdery mildew resistance locus to the 1RS-4 bin (corresponding to a ~50.68 Mb interval in the *Secale*_Lo7 reference), further fine-mapping is also required to narrow this interval to a candidate gene. This will involve developing a larger population from the available 1R translocation/deletion lines, screening with high-density markers, and ultimately identifying the target gene through functional validation. This novel germplasm will accelerate the cloning of candidate *Pm* genes for potential utilization in wheat resistance breeding programs.

## 4. Materials and Methods

### 4.1. Plant Materials

Wheat–rye double disomic substitution 1R (1D) + 4R (4D) line A156 and 1R (1D) substitution and 4R monosomic addition line A189 were developed by crossing hexaploid triticale lines Yukuri and T4915, respectively, with common wheat cultivar MY11, followed by successive backcrosses and selfing with selection by ND-FISH for the desired substitutions/additions. Yukuri was sourced from Seed Distributors, NSW, Australia [40]. The wheat cv. Mianyang 11 (MY11), Weining rye (WN; diploid rye), Jingzhouheimai (JH; diploid rye), Imperial rye (diploid rye), triticale lines 06-2-63 (hexaploid triticale), Currency (22W; hexaploid triticale), T701 (hexaploid triticale), Turum wheat-Secale africanum amphiploid YF (ABR^a^), the Imperial rye 1R addition line (CS-1R) and 1RS.1BL translocation lines were maintained at the Center for Informational Biology, School of Life Science and Technology, University of Electronic Science and Technology of China. Line A156 and line A189 were used as the female parent in crosses with wheat cultivars MY11, and two 1R (1D) substitution lines were developed from the F_2_ generation. To induce structural variants for fine mapping, seeds of the derived stable 1R(1D) substitution lines (R156 from A156 and R189 from A189) were irradiated by ^60^Co-γ rays at the Biotechnology and Nuclear Technology Research Institute, Sichuan Academy of Agricultural Sciences, China. The resulting M_0_ plants were crossed with MY11, and their progenies (M_1_-M_3_) were screened for 1R aberrations.

### 4.2. ND-FISH and Oligo-FISH Painting

The root-tip metaphase chromosomes of all germinated seeds were prepared according to the procedure described by Han et al. [41]. Synthetic oligonucleotides Oligo-pSc119.2, Oligo-pTa535, Oligo-ku, Oligo-K288, Oligo-pSc200, Oligo-V03-701, Oligo-StC12, Oligo-6E571 and Oligo-PtA1-360 were used for ND-FISH analysis [42,43,44], and their sequences are shown in Table S2. All oligonucleotide probes were either 5′end-labeled with 6-carboxyfluorescein (6-FAM) for green or 6-carboxytetramethylrhodamine (Tamra) for red signals. The pictures of FISH results under an Olympus BX-53 microscope (Olympus Corporation, Tokyo, Japan) were taken by a DP-70 CCD camera.

### 4.3. Chromosomal Immunolocalization

Rabbit polyclonal antibody anti-CENH3 was purified by affinity chromatography [45] and was produced by GL Biochem Ltd. (Shanghai, China). In addition, anti-phosphorylation AG3875 (mouse monoclonal antibody) and AG3665 (rabbit polyclonal antibody) from Beyotime Biotechnology, Shanghai, China, and anti-methylation ab8580 (rabbit polyclonal antibody) from Abcam, Waltham, MA, USA, were used to detect anti-CENH3 hybridization sites. Immunolocalization on mitotic metaphase chromosomes was performed as described previously [46]. The locations of anti-CENH3 hybridizations on chromosomes utilized CENH3 (1:300)/phosphorylation (1:500)/methylation (1:500) and goat anti-rabbit Texas green (1:1000)/goat anti-mouse Texas red (1:1000) (Sigma-Aldrich, St. Louis, MO, USA). The images were collected with the BX53 Motorized System Microscope (Olympus) and processed using Adobe Photoshop CS 4.0 (Adobe).

### 4.4. Molecular Marker Analysis

DNA of all materials used in this study was extracted from young leaves using the SDS protocol [47]. CINAU markers of linkage group 1 based on intron length polymorphisms were obtained from Zhang et al. [48]. SSR markers were obtained from Li et al. [49] The details concerning each of the markers are listed in Table S3. Physical locations of specific-1R markers were obtained using the database of the Lo7 genome on the website of the Triticeae Multi-omics Center (http://202.194.139.32/, accessed on 1 May 2018). The PCR amplification program involved 4 min at 94 °C, 35 cycles of 45 s at 94 °C, 45 s at 58 °C, 1 min at 72 °C and a final extension at 72 °C for 10 min. PCR amplification products were analyzed by 1% agarose gel electrophoresis and 8% PAGE gel electrophoresis, as described by Hu et al. [50] and Yu et al. [51], respectively. Information on the LRR resistance-like genes and receptor-like kinase genes was obtained from Steuernagel et al. [52] and Yin et al. [53].

### 4.5. Powdery Mildew Infection Scoring

The reactions of parent wheat MY11, line A156, line A189, two wheat–rye 1R (1D) substitution lines and their derived progenies to powdery mildew were assessed at adult-plant stages during the 2024–2025 season in a greenhouse. The inoculation was carried out using a mixture of locally prevalent *Blumeria graminis* f. sp. *tritici* (Bgt) isolates collected from infected wheat leaves of susceptible commercial varieties in Sichuan Province, China. These isolates were maintained and increased on susceptible wheat seedlings (cv. MY11) in an isolated greenhouse. The inoculation was carried out at the four-leaf stage in a greenhouse at 23 °C, and the response was recorded at 20 days post-inoculation. Infection types were scored according to the system described by Bariana and McIntosh [54].

## Figures and Tables

**Table 1:** Information on LRR resistance-like genes and receptor-like kinase genes.

Gene ID	Chromosome	Start Position	Functional Annotation	Expression After Infection
SECCE1Rv1G0013180.1	1R	101168790	LRR disease resistance protein	Yes
SECCE1Rv1G0013280.1	1R	101711741	LRR disease resistance protein	No
SECCE1Rv1G0013650.1	1R	107294565	LRR disease resistance protein	Yes
SECCERv1G0013500.1	1R	106151068	receptor-like kinase	Yes
SECCERv1G0010840.1	1R	66320586	receptor-like kinase	No

## Data Availability

The datasets used and/or analyzed during the current study are available from the corresponding author upon reasonable request.

## Supplementary Materials

The following supporting information can be downloaded at: https://www.mdpi.com/article/10.3390/plants15030410/s1, Figure S1: ND-FISH of mitotic metaphase of 1R (1D) substitution lines derived from Yukuri (a,b) and T4915 (c,d) by probes Oligo-k288 + Oligo-ku (a,c) and Oligo-pTa535 + Oligo-pSc119.2 (b,d). Scale bars, 10 μm. Figure S2: The anti-CENH3 location and ND-FISH of mitotic metaphase of 1R (1D) substitution lines derived from Yukuri (a,b) and T4915 (c,d). Immunostaining with anti-CENH3 (a,c) and sequential ND-FISH by Oligo-pSc119.2 + Oligo-pTa535 (b,d). Scale bars, 10 μm. Figure S3: The anti-phosphorylation AG3875 (a,d) and AG3665 (b,c) location and sequential ND-FISH by Oligo-pSc119.2 + Oligo-pTa535 (c,f) in 1R (1D) substitution lines derived from Yukuri (a–c) and T4915 (d–f). Scale bars, 10 μm. Figure S4: The anti-methylation location and ND-FISH of mitotic metaphase of the 1RS.1BL translocation line. Figure S5: PCR amplification patterns of Yr9 GSP markers. 1, MY11; 2, Yukuri; 3, T4915; 4, 22W; 5, CS-1R; 6, JH; 7, 06-2-23; 8, ABR^a^; 9, WN. Figure S6: Rye 1R chromosome variations from the M_1_ progenies of R156 × MY11. The probes were Oligo-ku + Oligo-k288 (left) and Oligo-pTa535 + Oligo-pSc119.2 + Oligo-pSc200 (right). Figure S7: The comparative PCR amplification results for (1) MY11, (2) T4915, (3) Yukuri, (4) A189, (5) A156, (6) R189, (7) R156, (8) 22W, (9) CS-1R, (10) JH, (11) 06-2-23, (12) ABR^a^ and (13) WN. The red and green arrows indicate the specific amplifications in Yukuri-1R and other 1R chromosomes. The primers were (a) CINAU882, (b) CINAU898, (c) SSR 1-082, and (d) SSR 1-124. Figure S8: (a–f) FISH of wheat–rye 1R derived lines X834 (a,b), X680 (c,d) and X861 (e–f). The probes were Oligo-k288 + Oligo-ku (a,c,e) and Oligo-pTa535 + Oligo-pSc119.2 +Oligo-pSc200 (b,d,f). The arrows indicate the 1R chromosomes. Bars, 10 μm. (g) PCR amplification patterns of 1R-specifc markers. 1, MY11; 2, Yukuri; 3, A156; 4, X834; 5, X680; 6, X861. The primers are Lo7_1R-1, Lo7_1R-2, Lo7_1R-3, Lo7_1R-4 and Lo7_1R-5 from top to bottom. Figure S9: Powdery mildew responses of A156, A189, wheat cultivar MY11 and their progenies at (a) seedling stages and (b) adult plant stage. 1, MY11; 2, A156; 3, R156; 4, individuals without 1R^Yukuri^; 5, A189; 6, R189; 7, individuals without 1R^T4915^. Figure S10: The ND-FISH results of 1RS.1BL translocation and rye 1R chromosomes from different accessions. The probes are listed on the right, and the ND-FISH fluorescent banding pattern map is shown below. Table S1: Responses of tested materials to powdery mildew at the adult stage. Table S2: The sequences of Oligo probes for 1R chromosome identification by ND-FISH. Table S3: The 1R-specific markers information used in our study.
