# Supplementary material for: Characterization of a New Powdery Mildew Resistance Gene on Chromosome 1R from Hexaploid Triticale Transferred to Wheat
Source: Plants (Basel). 2026 Jan 29;15(3):410. doi: 10.3390/plants15030410 (PMC12899970; doi:10.3390/plants15030410)
Supplement: Supplementary file 1 [file plants-15-00410-s001.zip › plants-3977235-supplementary.pdf]

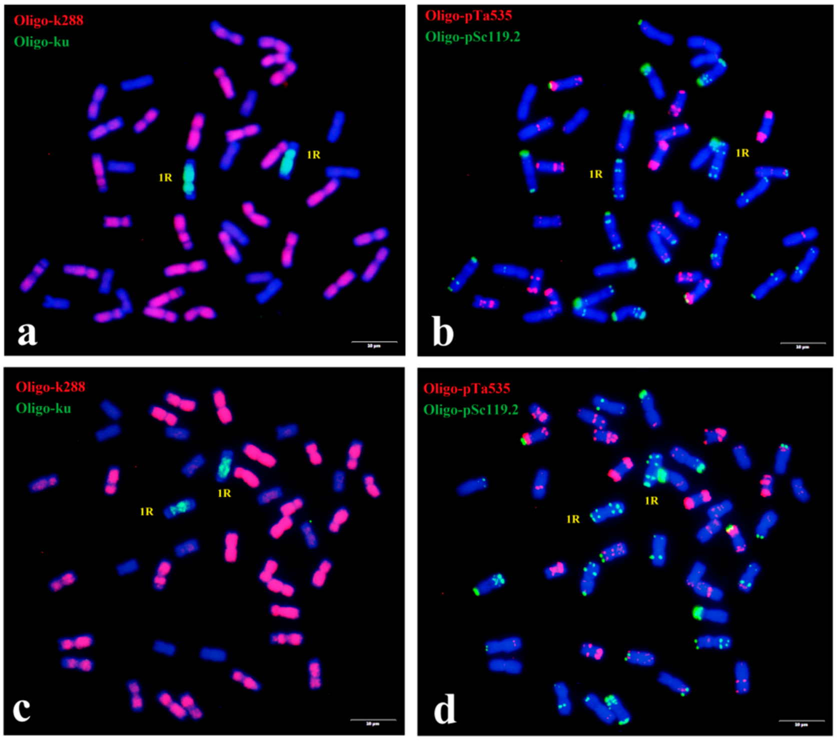

Figure S1. ND-FISH of mitotic metaphase of 1R (1D) substitution lines R156 (a-b) and R189 (c-d) by probes Oligo-k288 + Oligo-ku (a, c) and Oligo-pTa535 + Oligo-pSc119.2 (b, d). Scale bars, 10 μm.

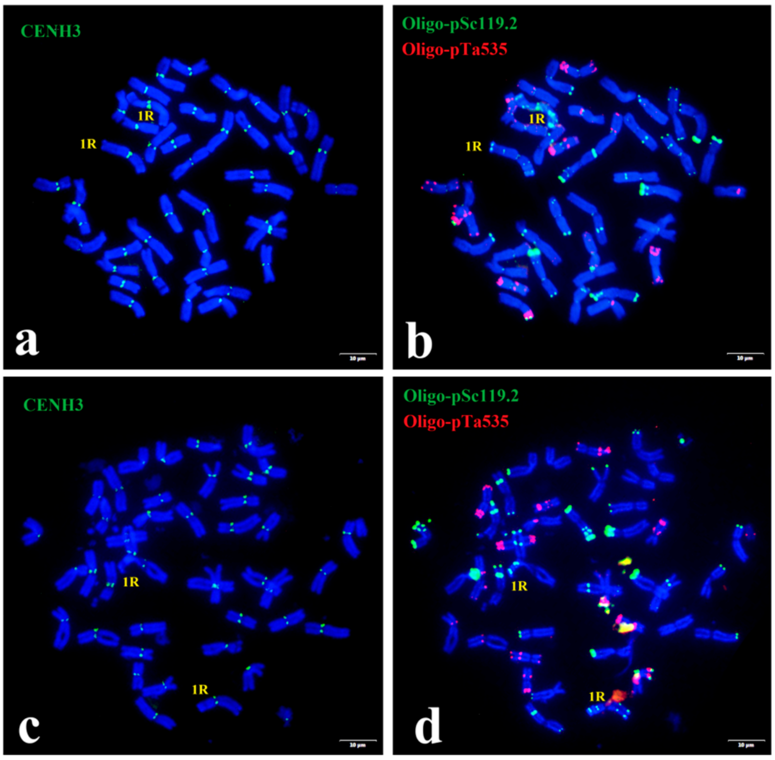

Figure S2. The anti-CENH3 location and ND-FISH of mitotic metaphase of 1R (1D) substitution lines R156 (a-b) and R189 (c-d). The Immunostaining with anti-CENH3 (a, c) and sequential ND-FISH by Oligo-pSc119.2 + Oligo-pTa535 (b, d). Scale bars, 10 μm.

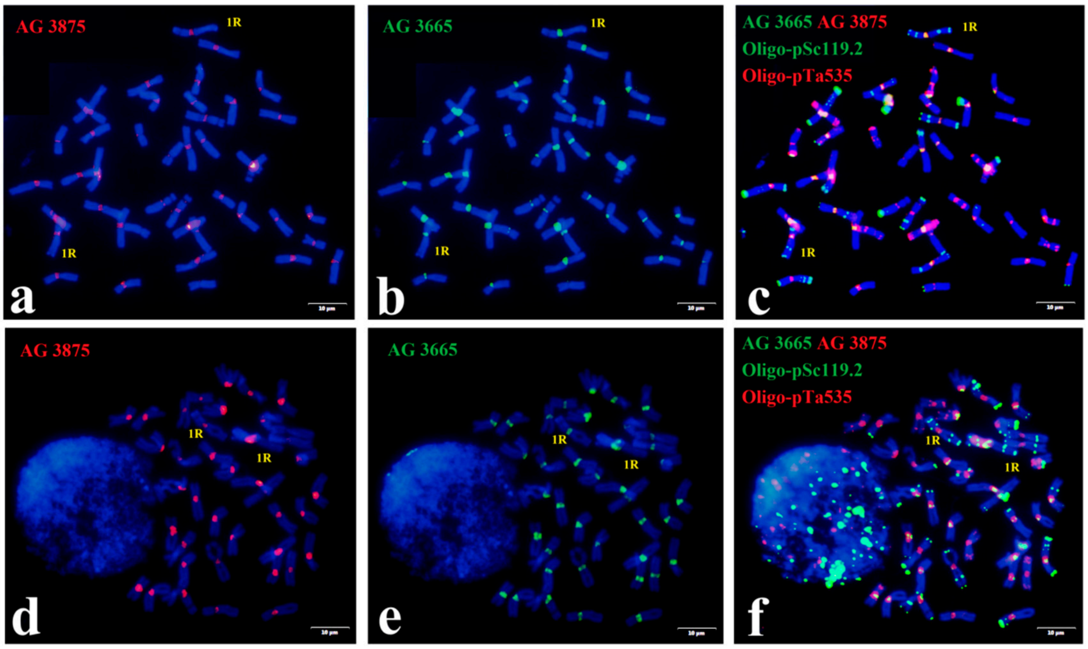

Figure S3. The anti-phosphorylation AG3875 (a, d) and AG3665 (b, c) location, and sequential ND-FISH by Oligo-pSc119.2 + Oligo-pTa535 (c, f) in 1R (1D) substitution lines derived from Yukuri (a-c) and T4915 (d-f). Scale bars, 10 μm.

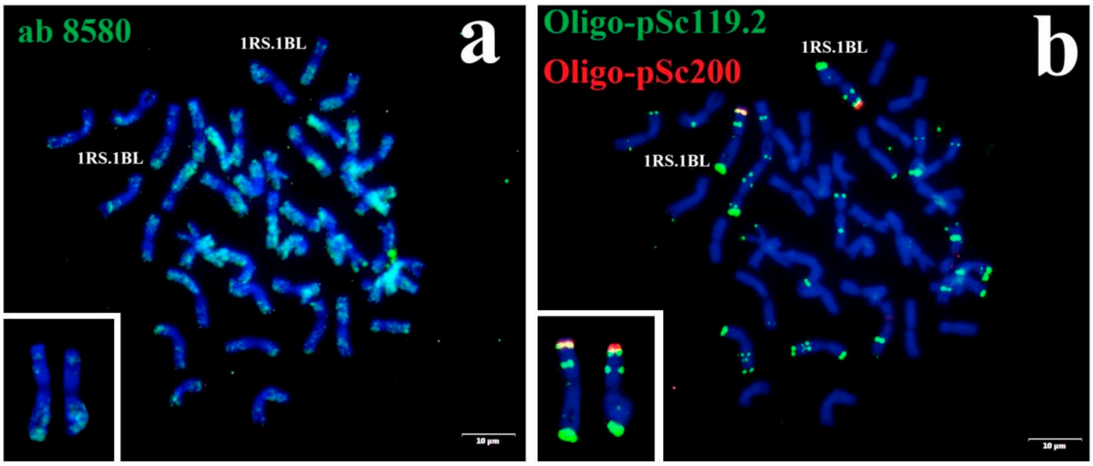

Figure S4. The anti-methylation location and ND-FISH of mitotic metaphase of the 1RS.1BL translocation line.

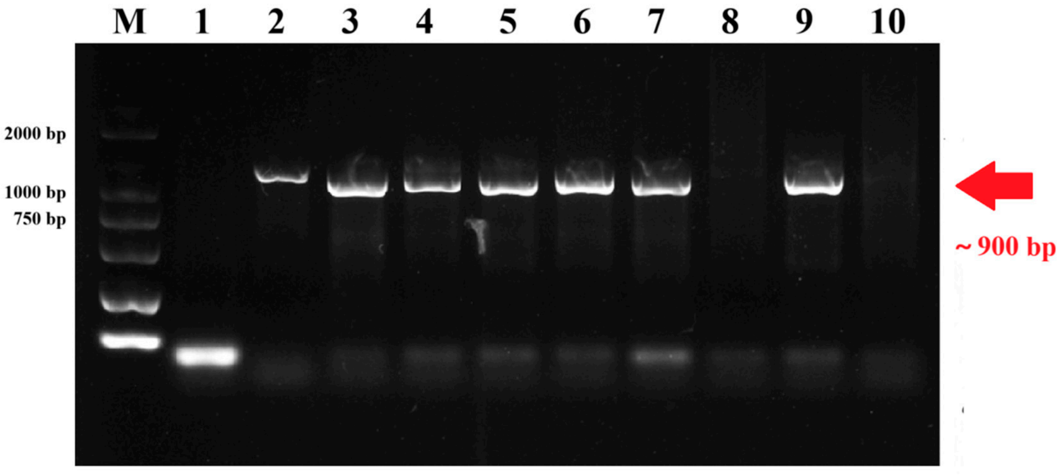

Figure S5. PCR amplification patterns of *Yr9* GSP markers. 1, MY11; 2, Yukuri; 3, T4915; 4, 22W; 5, CS-1R; 6, JH; 7, 06-2-23; 8, ABR[a]; 9, WN. Arrows point to *Yr9* specific bands.

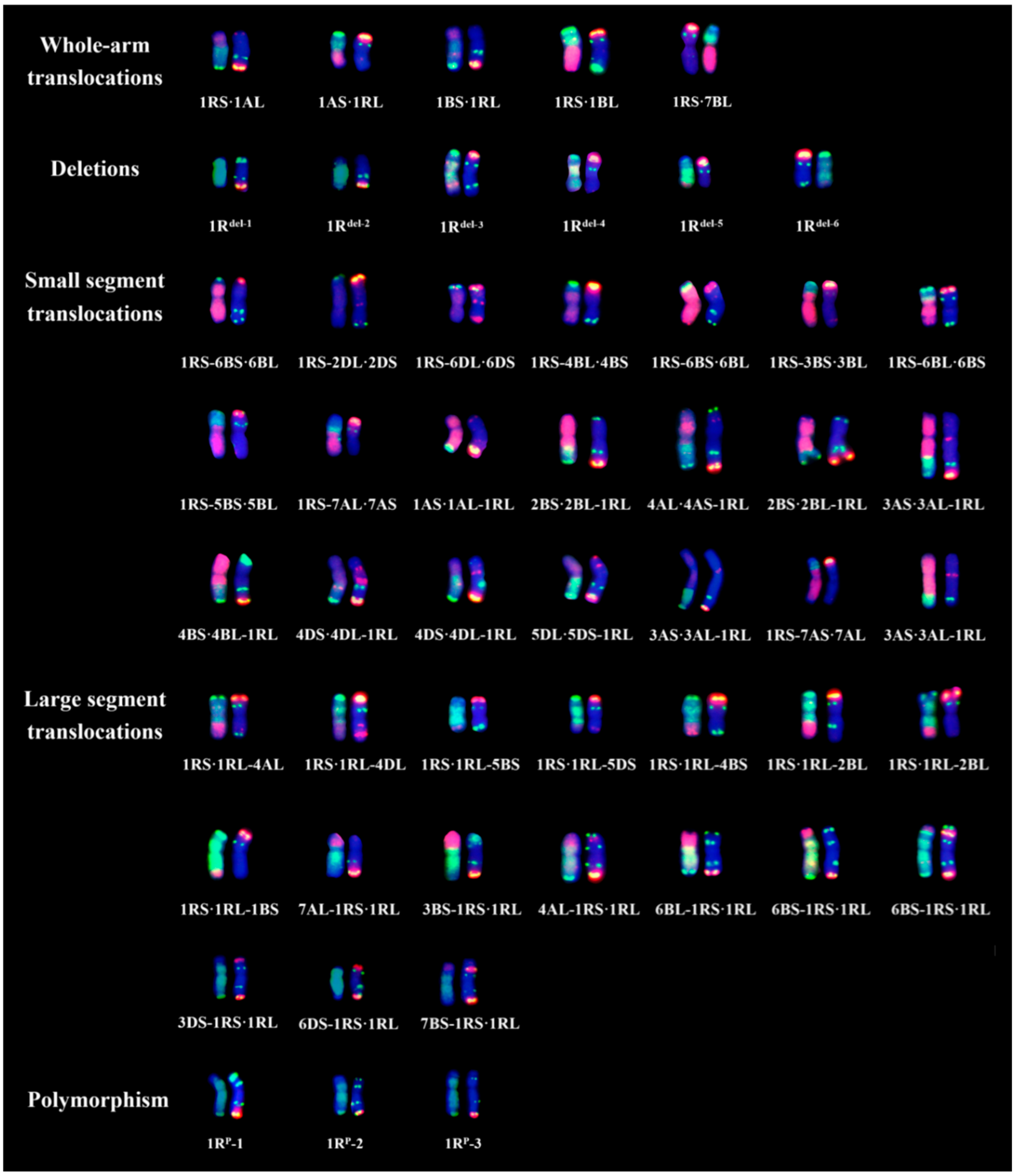

Figure S6. Rye 1R chromosome variations from the M_1 progenies of R156×MY11. The probes were Oligo-ku + Oligo-k288 (left) and Oligo-pTa535 + Oligo-pSc119.2 + Oligo-pSc200 (right).

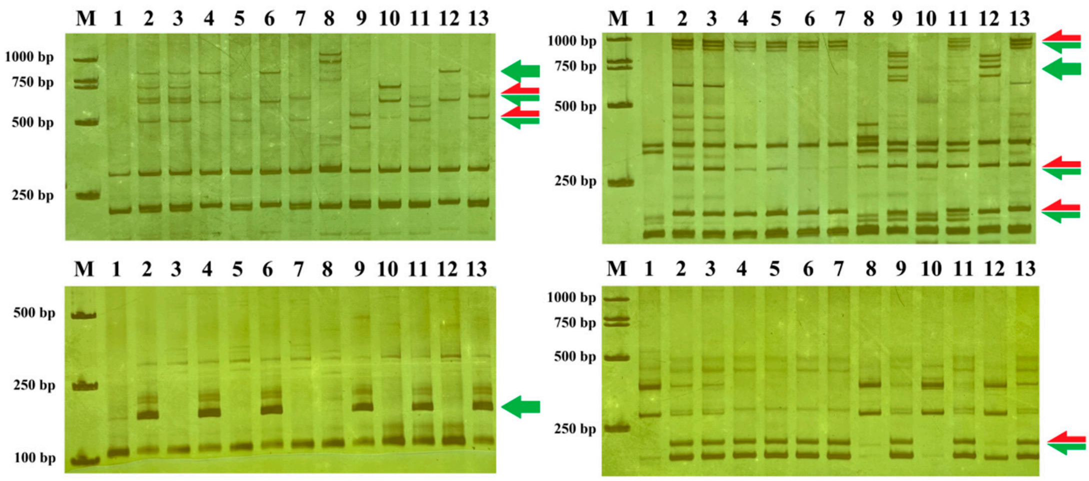

Figure S7. The comparative PCR amplification results for (1) MY11, (2) T4915, (3) Yukuri, (4) A189, (5) A156, (6) R189, (7) R156, (8) 22W, (9) CS-1R, (10) JH, (11) 06-2-23, (12) ABR[a] and (13) WN. The red and green arrows indicate the specific amplifications in Yukuri-1R and other 1R chromosomes. The primers were (a) CINAU882, (b) CINAU898, (c) SSR 1-082 and (d) SSR 1-124.

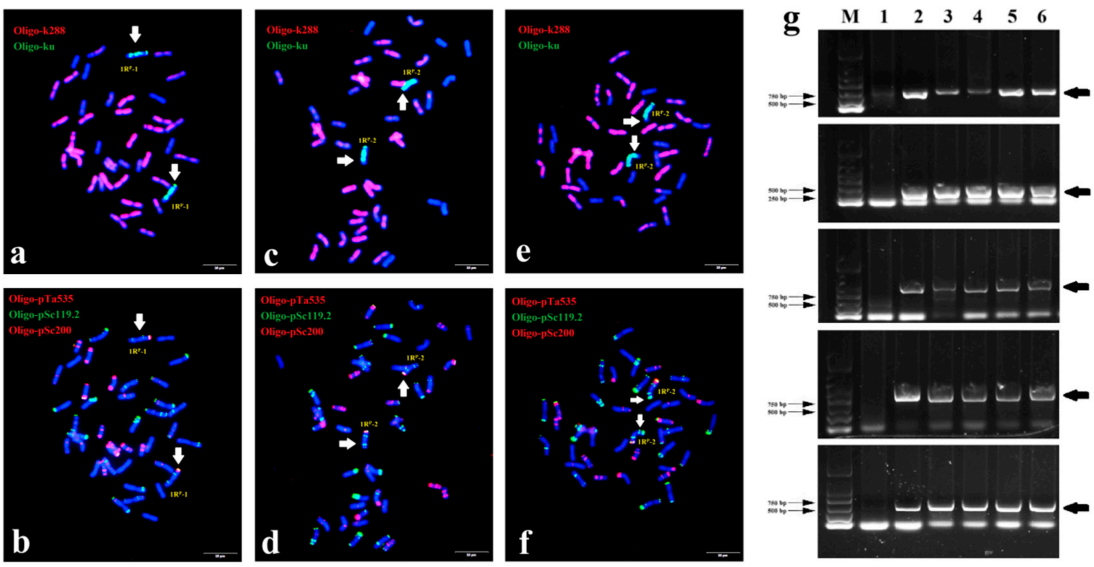

Figure S8. (a-f) FISH of wheat-rye 1R derived lines X834 (a-b), X680 (c-d) and X861 (e-f). The probes were Oligo-k288 + Oligo-ku (a, c, e) and Oligo-pTa535 + Oligo-pSc119.2 +Oligo-pSc200 (b, d, f). The arrows indicate the 1R chromosomes. Bars, 10 μm. (g) PCR amplification patterns of 1R-specifc markers. 1, MY11; 2, Yukuri; 3, A156; 4, X834; 5, X680; 6, X861. The primers are Lo7_1R-1, Lo7_1R-2, Lo7_1R-3, Lo7_1R-4 and Lo7_1R-5 from top to bottom.

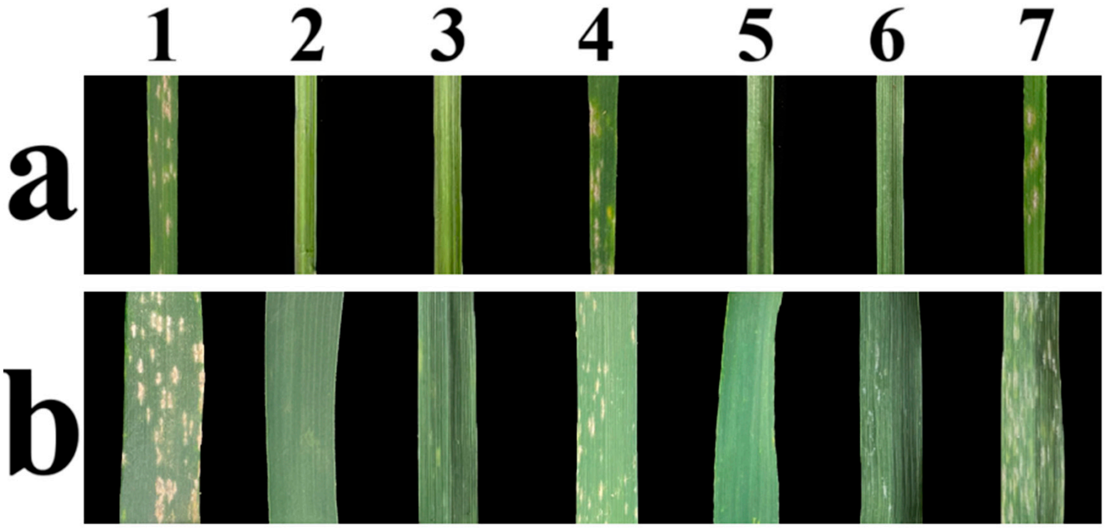

Figure S9. Powdery mildew responses of A156, A189, wheat cultivar MY11 and their progenies at (**a**) seedling stages and (**b**) adult plant stage. 1, MY11; 2, A156; 3, R156; 4, individuals without $1R^{Yukuri}$; 5, A189; 6, R189; 7, individuals without $1R^{T4915}$.

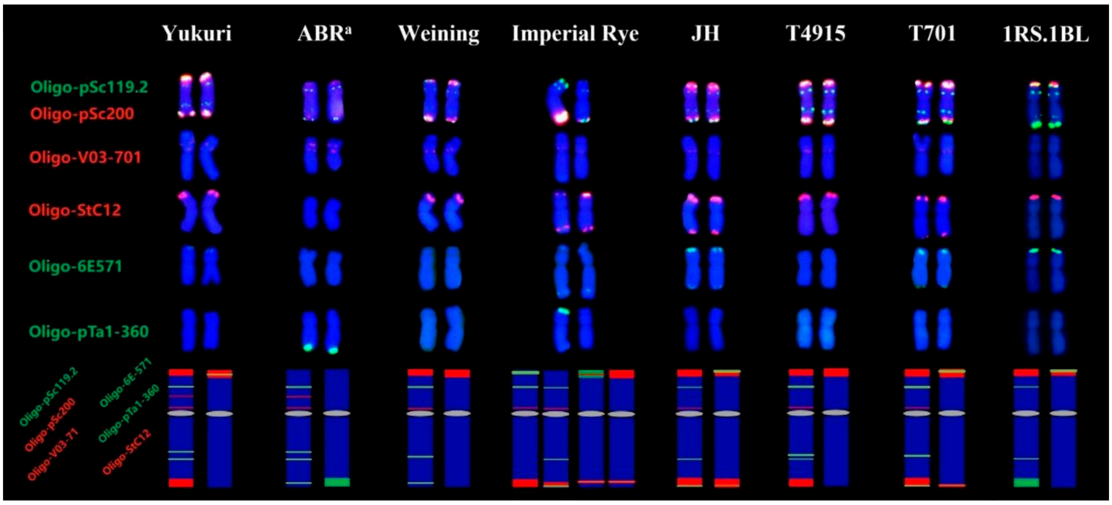

Figure S10. The ND-FISH results of 1RS.1BL translocation and rye 1R chromosomes from different accessions. The probes are listed on the right, and the ND-FISH fluorescent banding pattern map is shown listed below.

Table S1. Responses of tested materials to powdery mildew at the adult stage

Material Name	1R aberration type	ITs for *Bgt*
X814	3DS-1RS.1RL	0
X330	7BS-1RS.1RL	4

X617	6DS-1RS.1RL	2
X383	$1R^{del}$-1	2
X503	$1R^{del}$-2	4
X342	$1R^{del}$-3	0
Yukuri	Intact $1R^{Yukuri}$	0
T4915	Intact $1R^{T4915}$	0
A189	Intact $1R^{T4915}$	0
A156	Intact $1R^{Yukuri}$	0
R189	Intact $1R^{T4915}$	0
R156	Intact $1R^{Yukuri}$	0

Table S2. The sequences of Oligo probes for 1R chromosome identification by ND-FISH

Oligo probes	Sequences	Reference
Oligo-pTa535	AAAAACTTGACGCACGTCACGTACAAATTGGACAAACTCTTTCGGAGTATCAG GGTTTC	[42]
Oligo-pSc119.2	CCGTTTTGTGGACTATTACTCACCGCTTTGGGGTCCCATAGCTAT	[42]
Oligo-ku	GATCGAGACTTCTAGCAATAGGCAAAAATAGTAATGGTATCCGGGTTCG	[43]
Oligo-K288	TATTGATGATATGGGTAGTACAAGAGGAGATCTACCACGAGATCAGAGAGGCTA AACCC	[44]
Oligo-pSc200	CTCACTTGCTTTGAGAGTCTCGATCAATTCGGACTCTAGGTTGATTTTTGTATTTT CT	[25]
Oligo-V03-71	CTTCTCCGAGTGAAGAGCGATCCTCTCACTCGGGGGCTTAGCTGCGAGTCTAAT C	[26]
Oligo-StC12	ATGTTCTATCACCCATCCCGCTACCACCCTCACTTTTTCACCAG	[26]
Oligo-6E571	ACGTAACGATTTTAGAGGCTAGGAGTGGACACGCCAGCGATTAAATCATC	[26]
Oligo-PtA1-360	CCTTCAAAAGGAAGTGCCAATGGGTTCAGAAACTTGTCCGAAAAACAGCGGCG AATG	[26]

Table S3. The 1R-specific markers information used in our study.

Primers	Forward primers (5'-3')	Reverse primers (5'-3')	Location (bp)
Lo7_1R-1	CATGTGCCTACCAATTCTA	TACGCCCTAAACTTCTGTC	794294
Lo7_1R-2	TCTGTTCATGCCAGCCAC	GCTATTACATCTTCGGTCGG	1358151
Lo7_1R-3	TTCAATAGTTGCTCCCATG	GTCATAGGAAGCTGGTAGT	2299083
Lo7_1R-4	CTTGGAGCCTTACCCTTTC	TAGGGAAGCTGATTTTCG	102038404
Lo7_1R-5	CGGATGTAACTCACCAGC	GACGCATTCGGAAGGCTACTA	109280233
glk.1R-2364	AGTTTAGCCCAGCTCAAGCA	CTGCCGTTTGAATTGAAGGT	13691168
glk.1R-0728	GGTGCACTCTTCTTGGAAGC	ACGGTGGATCTATCCGTTGA	24620345
glk.1R-2740	TCTCATGGGATAGACGGAGC	GGCCATAAATGACAAATGGG	32447652
glk.1R-4306	GTTTTGGGAATATGGCATGG	GACGCACCAATTTCTTCGAT	41351133
glk.1R-1728	ACGTATGACGTCGGCGAGTA	GAACGACGCCCATCTTGTAT	53551016
glk.3R-5062	GTTTAGGGGTGGATCGGATT	GGTGAGGGAGATTCATTGGA	161287408
glk.1R-1703	TATTCCATGGAGTGGGGAAC	TTTTTGCAGACACATCCAGC	186503836
glk.3R-4318	ATGGACCGTAGTTGGGTGAG	ATGGACCGTAGTTGGGTGAG	480302466
glk.3R-4028	GTAGCCATGCTTGGTTCCAT	CGTGAGAGAGGGAAGGAGAA	213212326
glk.1R-1551	ATCAACGCAGCCGTTATTTC	TATAATCCCTCCCCAGACCC	254426565
glk.1R-0961	CCCTGCTGGTTTCTGATGTT	GTGACAACACGAATGCAATG	296780698
CINAU836	AGCCCCTCTTCATACATTGTCA	CCCAAGAAGACGGCCAATAA	298406578
CINAU838	TGCCTCAAATGTTCCAACCG	CGGATGCTGCCACTCTTGT	595959990
CINAU843	CCTGTTATGGTGCGGAGGA	GGCCTCCCTAACTTCTTCACA	552901804
CINAU846	TGATTGGGCAGTATGAGGCA	CCTTCACACTTCCCAATAGCA	567334863
CINAU847	ATGGCTCCTTCACTACGAGC	ACGTTTCTGATCCAGCTCTATTG	359046399
CINAU851	TCTTCGTATCAGGCTTTGCTT	ACCTTGCGGATCCTTCTCAA	317183490
CINAU852	CCATTTGGGTTTGTCGGAGA	CGAAGGTACGGAATGTTCTTTTG	591692173
CINAU853	AAAAGAACGAAGCACCGACC	GCTCCTCCGCTGTCTTTCT	389986693
CINAU855	TCTGCAAAGATCGATACAAGGT	CTGCAATTCTTGTGCCTCCA	41919940
CINAU856	ATTCGTGGGTGCTTGGAAGA	TGAAGACCATGATTGCCACC	375977994
CINAU863	CTCCCTGAGGTAGTGGAAGC	CGAAGTAAAAGACCAGCAGCA	442814118
CINAU866	TCCAATCATTGCGCCAAATCT	GCAGTGTCCAAAAGTCCCTT	369115071
CINAU867	TGAAGGCCACACTGAAAGGA	GGATCGTCGGACCTCTCAAA	303450015
CINAU868	CCAAGGAGAGGGAGAGCG	AACAAGTCTTCCCTGCGCC	495397472
CINAU869	TGATATGATTGGCGTTGATGC	AGTCGGTGTGAAAGGACCTC	483698275
CINAU884	TGGCAATGTCTGTAGCCATC	GAACTCAAGCCGTGTCATGG	384205224
CINAU880	CAGTTCGCGCAAATCTACCA	CCGTTGGAAGTTTTCAGTTGC	569251871
CINAU882	GACCCAGATATACGCCAGGA	GATCTGCTCCTTGGACTGGT	446828508
CINAU887	TCCAGATGCCAAGGGAGAAG	ACACAGGCACATCATCAGGA	578261229
CINAU892	AAGGCAGGGCTTTGTCAAAT	TGTTTGCCTGCTAGAAGTCTTC	495769738
CINAU896	GCTCATGGTGTTTGCTTAGGA	TGTATAGTCCGTGCAATTCATCT	644774468
CINAU898	ATGGACGCCTCGCCTCTC	CTCCCGTAGCACACCAGC	417172141
CINAU899	CGTGCCATGCTTATTCAGGT	TGCCTTGAGAATCTGATCCAG	694121612
CINAU902	ATCCATGGCGCTTCAGATAG	GACCAGAGGAGGAAGGCG	459511212
CINAU909	AGCAGCTTGGAGAACTTGGT	TCCCGAGGTATCAAGACTGC	658094943
CINAU911	AGAAAACAGTTATCGTGGATGCT	CAAGTCATGCCATGGTTCCC	335380542

CINAU912	AGAAAGCTTGTTTGGTTGGTCA	GCCACTGTTGACGAGCCTAT	673546336
CINAU917	GGTGCTCCAGGGAAGTATGT	GCCGTCCAAAACACCCTG	710528117
CINAU919	GCAACTTCCAGTGGTTTCGT	GCCTTATGCCTTAAAGCCGA	640116677
CINAU920	GGAACGTTGCAAACCTAGGA	TACCCCGCTACTTCAACTGG	719303666
CINAU922	GGAGTGATCCAGCTGCCC	TAGCTTCCCTGACCTTTCGG	46709039
CINAU940	GAGCATGTCTGAGTTGGAGT	CCCCTATGAAGCCCAAAACG	20326948
CINAU942	ACGTTCACTCCAAGATGATGA	CTCGTTGAGGTTGGCAGATC	139027200
CINAU943	AGAGAACTGAAATCACTGAGGGT	CACCCTGTCAGCATGAAGTG	160013186
CINAU945	GATTCGCCGAGATTGTTGTCA	ACCGGTAGAAATCCTGAGGG	123302929
CINAU948	CCTTGTTGAGTTCTATGCCCC	GCAATCACGACACCCTCATC	191583009
CINAU949	GTTAGACATTGGGGAGGGCA	ACATAAGTTTGCCACCCTCA	17260636
CINAU953	CTCAAGCCTCAGTTTTAGCCC	ATCTCCCAACACCTCACCTG	240951401
CINAU957	TTGCCTTCTACAACATTCCGA	TACCAATCTCTGCTGTCCCC	91132593
CINAU959	TCATCTGCCGAGGATCAATAGT	TGTAGGCGTATCAGTTGGCT	187206746
CINAU961	CTCTCCGTTGGTAGCTGTG	AGTAGAAAGGCATGAACTCCAC	6195259
CINAU967	AGGAAGATGGGCCAAGAGAA	TCGACCAATCTCTGCACAGT	196808217
Xglk.ssrR1-037	GTGCAGCGAGGAGAAGAACT	CTTGCAAGCACAAGCAGCTA	207252178
Xglk.ssrR1-040	TGCAAAACTTGAGAGAGGGAG	ATGGAGGAATTGCTGGGTAA	566792594
Xglk.ssrR1-047	GTGGAGAGGAGTGCGAACTTA	GTTAGTTGTTCAGGCGCAGC	224621561
Xglk.ssrR1-059	AGAACCACCTCAAATCGGTG	GGCCCTGTTGTAATTTTCTCC	185534514
Xglk.ssrR1-061	GTGGCACTCTACGAGGTTGA	CGGACAAGTGCACTGTGAAA	672058212
Xglk.ssrR1-072	CAACGACAGCTCGTGCATAC	TGGTTGACCTGTTCCATCAA	691292248
Xglk.ssrR1-082	TGTGCGTAGCTAGTCGAGGA	GCCCGTTTGATCACTTTCTG	213238291
Xglk.ssrR1-087	AAAGAAGACGACCCAAGGTG	TCCAGACCATCTGCAAAAGA	221620768
Xglk.ssrR1-091	CTTAACATGCCCCGAAGAAG	CACACCACCAGAACAGAGGA	562771328
Xglk.ssrR1-093	ACCGGGAAGACTTTGGTTTT	TTTGGTTCCACATCATCTCG	562731775
Xglk.ssrR1-096	GCCCACCATGTTTTCCTTTA	GGATATGCGTGTGTGCTATCAT	287104507
Xglk.ssrR1-103	ATATGTCACCGATTCTCGCC	CAGCCGCTACAGTATCCGTTA	153926844
Xglk.ssrR1-106	GGGTGTAGAGAAAGAAGAGAGGG	AAAAGGATGAGCAACGATGG	317725791
Xglk.ssrR1-109	ACCCTCCTCAAATATCGCCT	AAACGGTTTGTTGCCCATAG	64522836
Xglk.ssrR1-121	GAAAGTGCAATCACGCCTTA	TCTTGTCGCTGTTGCACAAT	297095008
Xglk.ssrR1-124	TCGAGACCTAAGTTCGAGGG	CCAAGGTGCCTTGTAAGACTG	4297627
Xglk.ssrR1-126	GGTAGGTAGAGGAGGAGGAGATA	GACCCTCGACGTTGAGACAT	655875325
Xglk.ssrR1-131	TGATTACGCGCTGTTAGTGG	AAGGAGACGAGCAGAAGAAGAA	17405234
Xglk.ssrR1-132	ACGAGGTTGGTGTTGGAGAC	GCATGCGGGTACTAGCTGTT	17405234
Xglk.ssrR1-137	GGTGCACTCTTCTTGGAAGC	ACGGTGGATCTATCCGTTGA	24620345
Xglk.ssrR1-138	AGGGGAGAGGGAGAGAAACA	CCCCAACTAGTGACAGCAGA	48391800
Xglk.ssrR1-144	TTTAAGCAAACGTGTGCCAG	TTCTGGCTTGTCTTGTACTCCA	705534860
Xglk.ssrR1-155	GGCAAAGTATCCCAACAGGA	GAACATAGTTTTCGCGCCAC	679466615
Xglk.ssrR1-156	GGCGTTGGTTGTAGCATTTT	TATTTTGCTACCGCCGTCTT	390164550
Xglk.ssrR1-164	CTCCATTCCTCTCCCTATGC	AATGCGTGTATCGGATCTGG	424597503
Xglk.ssrR1-166	GATTAGTCCAAACGAGCGGA	ACAAGGCATCGGTCAAGAAG	604932977
Xglk.ssrR1-186	TGGAAAAGCAGAGTGAGAGGA	ATGAAGCGATCGAGGGAAGT	496577871
Xglk.ssrR1-188	CTTCAAGCATTGCCCTCCT	CTCGTTGAGGTTCATCCTGC	719156694

Xglk.ssrR1-190	ACTGCACACCTGCATAGTGG	TGTTCCAGGAAACTCATCGG	345835787
Xglk.ssrR1-193	GAGAAGGAGGTGGGAACACA	ATAGAAGCAATGGCCAGGTC	643145437
Xglk.ssrR1-204	TATGAGAGGTGTGTGCGAGG	GGCAATGATAGGCCTAGAACC	273504252
Xglk.ssrR1-206	TGTAAGCAAGCGTCGTCATC	TAGACCACTGCTCGAAACCC	437601275
Xglk.ssrR1-208	AGGGGAGGGTGTTAGGAGAA	TTTTCGATGTGGCAGTTAGC	431567140
Xglk.ssrR1-221	AGCAATTGCGAAGTTGACCT	GGAAGAAAACAACTGTCACTGC	592460761
Xglk.ssrR1-232	GCTCTAGGAAATCGGGATGG	GGCCGACTCTGTCGAGATTA	127136037
Xglk.ssrR1-233	TCGAGTCTCTCATGCAGTGG	TGGCGGGTCCATAATTGTAT	55025563
Xglk.ssrR1-236	GATGCTGGAGAGAGTGGCAT	CCTCATTGCGTTGATGACTG	103224327
Xglk.ssrR1-237	GGGGCACCAGCTCAATAATA	CAACAAGAGCGCGACACTAT	505543763
Xglk.ssrR1-249	AATGGGGAGGGGAAGAGAAT	ACCTCCTCACTGCGTCATCT	344208629
Xglk.ssrR1-250	CGACGTTGCGTGCTAGTATC	ATTTGAGCTAGGCCGTGAGA	163823788
Xglk.ssrR1-255	ATCACTCCCTTTTGCACACC	TGGTTAGGGAGAGTTGCACA	529812908
Xglk.ssrR1-265	TCACTTAGGCATAGAGTCCCA	GAGCCTCTCTCATCGAATGG	332817239
Xglk.ssrR1-268	GCTCTCCTAGGATCCATATTTCC	ACCTCACGATACCGTGCTTA	236169928
Xglk.ssrR1-270	AAATGAAGGTGCCAAACAGC	GGAGGCAGTTCTTCAGGACA	176840725
Xglk.ssrR1-274	CCCAATTCTCACGCACAAT	CTTCTACCAAACGCGTAGCC	207252067
Xglk.ssrR1-277	GGCACGAGCAAGAGGAGATA	TCATTTGTAACCTGGGAGGC	379314967
Xglk.ssrR1-283	CGGTGTTGTGAACCTCCTTT	GTTCCCACATGTTGTCTCCC	58600185
Xglk.ssrR1-286	TCGACCCCTTCAAAAGATTG	AAAAGCATCTGACACGTCCA	486725874
Xglk.ssrR1-289	GCGTGTTTGGCATTGTTTCT	CACTTGGTCTCCATTGGGAT	718652966
Xglk.ssrR1-301	CGGAACACCCCATGTATTTC	GGTGTGGAATTTTCTCGTCC	255213352
